# High glucose concentrations induce oxidative stress by inhibiting Nrf2 expression in rat Müller retinal cells in vitro

**DOI:** 10.1038/s41598-022-05284-x

**Published:** 2022-01-24

**Authors:** Jesús Silvestre Albert-Garay, Juan Rafael Riesgo-Escovar, Rocío Salceda

**Affiliations:** 1 Departamento de Neurodesarrollo y Fisiología, Instituto de Fisiología Celular, Universidad Nacional Autónoma de México, 04510 CDMX, Mexico; 2 Instituto de Neurobiología, Campus UNAM Juriquilla, Universidad Nacional Autónoma de México, 76226 Querétaro, Mexico

**Keywords:** Cell biology, Neuroscience, Diseases

## Abstract

Diabetic retinopathy (DR) is a complication of diabetes. Several studies have implicated oxidative stress as a fundamental factor in the progression of the disease. The nuclear factor erythroid-2-related factor 2 (Nrf2) is one of the main regulators of redox homeostasis. Glia Müller cells (MC) maintain the structural and functional stability of the retina. The objective of this study was to evaluate the effect of high glucose concentrations on reactive oxygen species (ROS) production and Nrf2 expression levels in rat MC. MC were incubated with normal (NG; 5 mM) or high glucose (HG; 25 mM) for different times. Incubation with HG increased ROS levels from 12 to 48 h but did not affect cell viability. However, exposure to 3 h of HG caused a transient decrease Nrf2 levels. At that time, we also observed a decrease in the mRNA expression of Nrf2 target genes, glutathione levels, and catalase activity, all of which increased significantly beyond initial levels after 48 h of incubation. HG exposure leads to an increase in the p65 subunit of nuclear factor-κB (NF-kB) levels, and its target genes. These results suggest that high glucose concentrations lead to alteration of the redox regulatory capacity of Nrf2 mediated by NF-kB regulation.

## Introduction

Diabetes mellitus is a metabolic disease characterized by increased blood glucose levels (hyperglycemia) resulting from defective insulin secretion or resistance to its action^1^. Diabetic retinopathy (DR) is one of the most severe complications of diabetes and is a leading cause of blindness in adults^2^. The precise mechanisms involved in triggering and the early progression of DR are still unknown, but it has been hypothesized that hyperglycemia causes oxidative stress^3–5^, which in turn leads to oxidation of biomolecules^6,7^. Indeed, hyperglycemic conditions increase the generation of reactive oxygen species (ROS), and the oxidation of retinal biomolecules of diabetic patients and long-term diabetic animals^6,7^.

Nuclear factor erythroid 2-related factor 2 (Nrf2) is one of the primary regulators of cellular redox homeostasis. Nrf2 mediates basal and induced transcription of phase II antioxidant enzymes responsible for the clearance of ROS^8^. In normal conditions, Nrf2 is sequestered by its binding to the Kelch-like ECH-associated protein 1 (Keap1) in the cytoplasm. Keap1 is a substrate adaptor protein for Cullin 3-RING-box protein 1 ubiquitin ligase and continuously targets Nrf2 for ubiquitination and its subsequent degradation by the 26S proteasome^9^. Several Keap1 cysteine residues are oxidized during oxidative stress conditions, causing a conformational change in the protein, and as a consequence, the release of Nrf2 allowing its translocation to the nucleus^10^. Nuclear Nrf2 heterodimerizes with one of the small Maf proteins (avian musculoaponeurotic fibrosarcoma homolog proteins). The Nrf2-Maf heterodimers recognize antioxidant response elements (AREs), promoting the antioxidant genes transcription^11,12^. Nrf2 drives transcription of hundreds of genes, encoding enzymes involved in antioxidant defense, as well as in glucose and lipid metabolism^13^. Loss of Nrf2 has been shown to exacerbate oxidative damage in retinas under different stresses^14–17^. Moreover, Nrf2 levels alterations have been reported in diabetic animal retinas^18–21^.

Although most DR complications have been ascribed to vascular lesions, various studies in diabetic patients and experimental animals suggest alterations in neuronal and glial functions before vascular abnormalities^22^. Müller cells (MC) are the principal macroglia type of mammalian retina. These cells extend from the photoreceptor cell layer to the ganglionar cell layer, contributing to retinal homeostasis by regulating synaptic activity, and providing energy metabolites and trophic factors^23–26^.

Starting at early diabetic stages in the rat retina, MC undergo a gliosis process in which they overexpress glial fibrillary acidic protein (GFAP), inducible nitric oxide synthase (iNOS), and pro-inflammatory proteins such as VEGF^23,27–29^. Several studies have reported an increase in cell death in MC exposed to high concentrations of glucose (25–30 mM) for long periods of time (48–96 h)^30–33^. Cell death has been associated with inactivation of AKT^33^, increased phosphorylation of insulin receptor substrate (IRS)-1^32^, and accumulation of glyceraldehyde-3-phosphate dehydrogenase (GAPDH) in the nucleus of MC^31^.

However, the effect of hyperglycemic conditions on the MC redox state had received insufficient attention. Therefore, we studied the time course of high glucose concentrations on ROS production and Nrf2 expression levels in cultured MC.

## Results

### Effect of high glucose on cell viability

**Figure 1:**
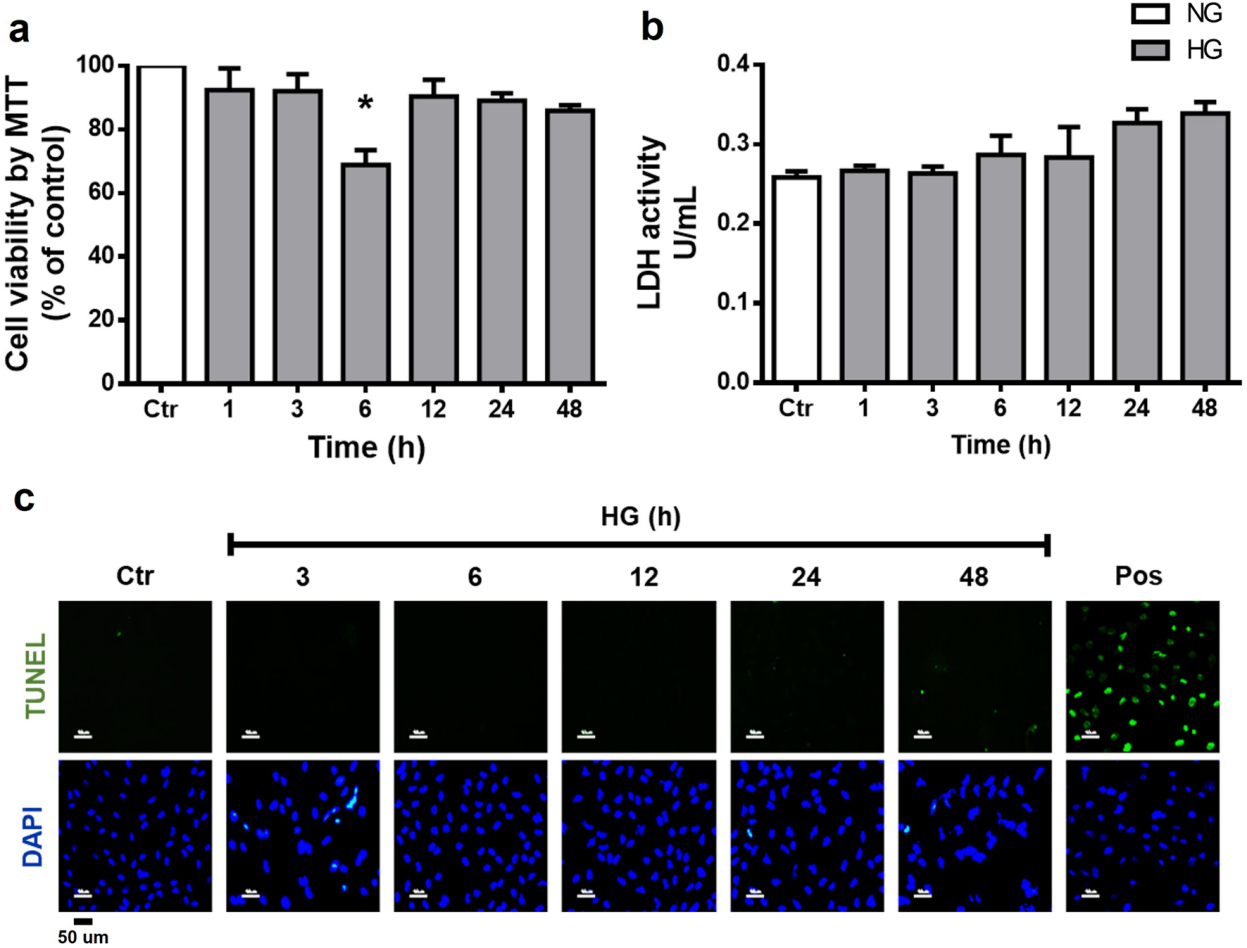
High glucose concentrations do not affect cell viability in MC. Cells were incubated with NG (Ctr; 5 mM glucose) or HG (25 mM glucose) for different time periods (1–48 h). (**a**) Cell viability was determined by the MTT assay; viability was expressed as the percentage of optical density respect to cells exposed to NG (100%). Cell death was measured through LDH release and TUNEL assay. (**b**) The results of LDH release are expressed as units per milliliter (U/ml). (**c**) Representative fluorescent micrographs of TUNEL-positive cells (green) compared to total cells (DAPI, blue) in MC exposed to NG or HG; positive control (pos: plus DNAse). Data are expressed as the mean ± SEM of duplicate cultures and are representative of three independent experiments. Scale bars: 50 µm. *p < 0.05 with respect to NG.

We first evaluate the cytotoxic effect of HG concentrations on MC using the MTT, LDH release, and TUNEL assays. As shown in Fig. 1, cell viability was not significantly affected by HG incubation. Cell viability determined by the MTT assay was not significantly affected by HG incubation, except a significant decrease in cell viability at 6 h incubation (68.9% ± 4.6; Fig. 1a). Similarly, LDH release did not show significant changes over the times studied (Fig. 1b). The TUNEL assay was also negative at all times examined (Fig. 1c).

### High glucose concentration increases ROS and RNS production in Muller cells

**Figure 2:**
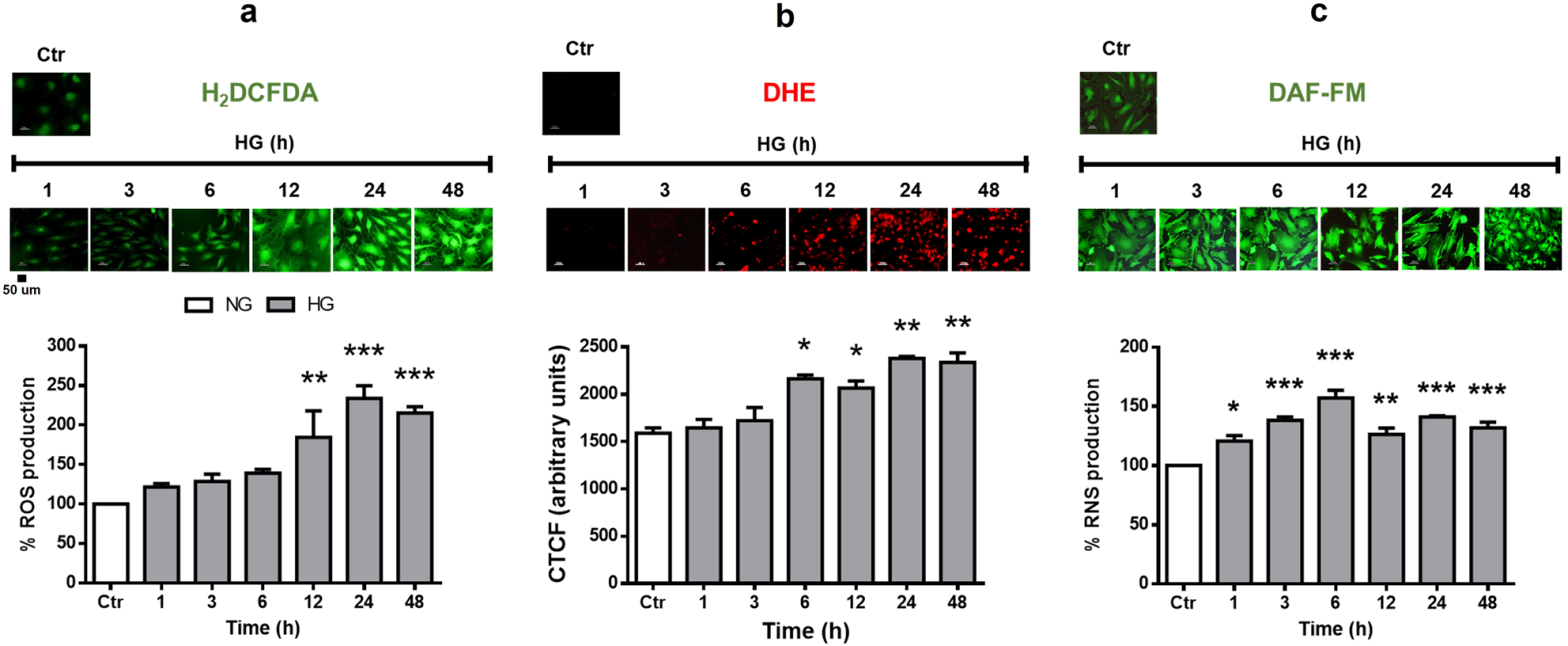
High glucose concentrations increase ROS and RNS production levels in MC. Cells were incubated with NG (Ctr) or HG for different time periods (1–48 h). (**a**) Production of ROS in MC. In the upper part, representative fluorescent micrographs. The lower part, the percentage of relative H_2_DCFDA fluorescence per µg protein. (**b**) Representative micrographs of DHE-positive cells (red). (**c**) Production of RNS in MC. The upper part, representative fluorescent micrographs. The lower part, the percentage of relative DAF-FM fluorescence per µg protein. Data are expressed as the mean ± SEM of duplicate cultures and are representative of five independent experiments. Scale bars: 50 µm. NG, normal glucose; HG, high glucose. *p < 0.05 with respect to NG, **p < 0.01 with respect to NG, ***p < 0.001 with respect to NG.

We investigated the effect of HG on cellular ROS levels. MC had low ROS production under normal glucose concentrations, as revealed by the H_2_DCFDA probe and DHE staining. Under HG incubation, a significant increase in ROS production determined by H_2_DCFDA was observed at 12 h (160% ± 33.8), with levels remaining constant up to 48 h (212% ± 8.1; Fig. 2a). Likewise, DHE staining was observed after 6 h incubation in HG (2190 CTCF ± 40.1), and its intensity increased over incubation time (Fig. 2b). On the other hand, RNS production was relatively low under low glucose concentrations, but was significantly increased after 1 h incubation in HG (140% ± 1.0) and remained high through all other times studied. (Fig. 2c).

### Nrf2 expression in Müller cells

**Figure 3:**
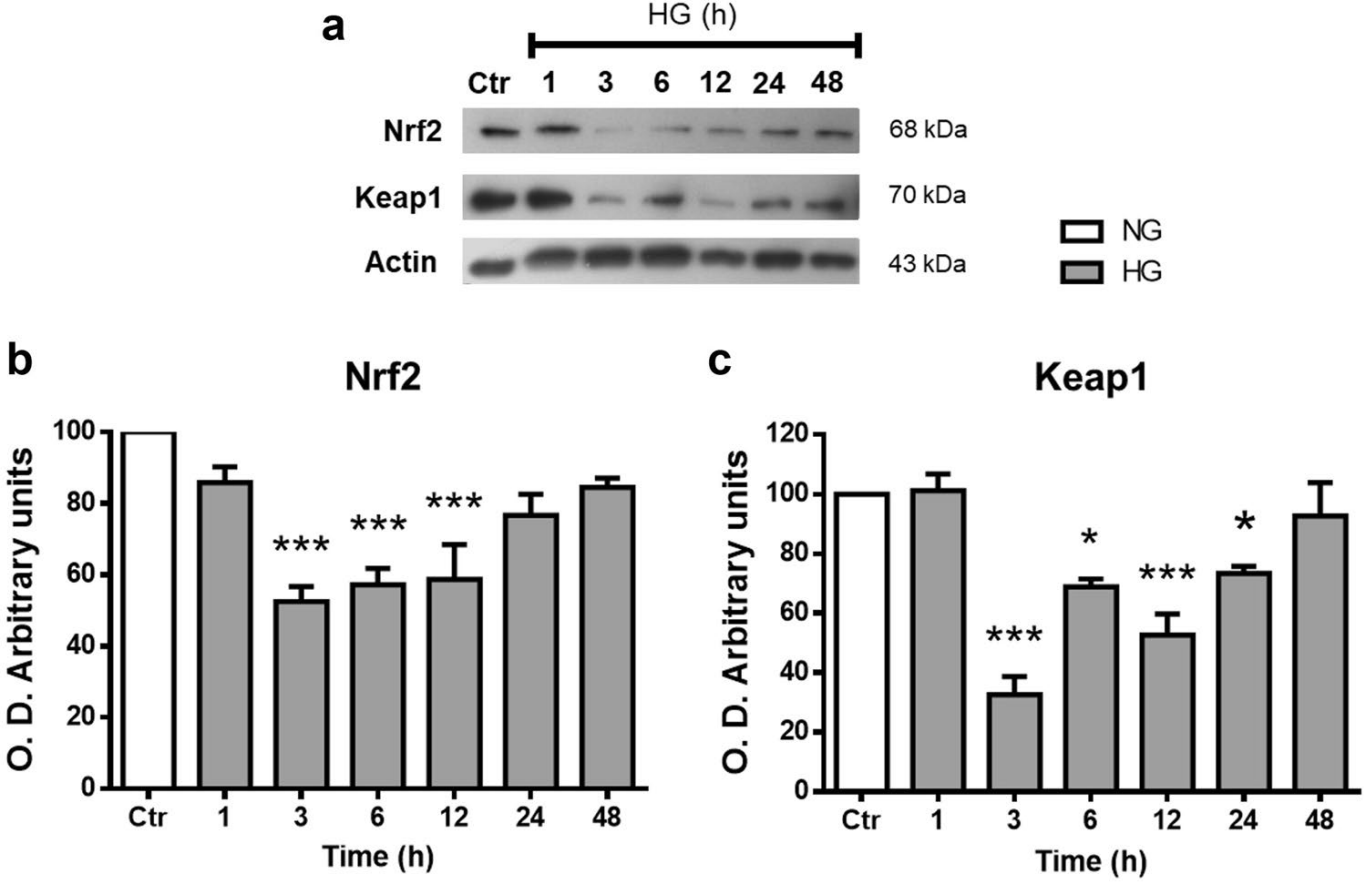
High glucose concentrations decrease Nrf2 and Keap1 levels in MC. Western blot analysis of Nrf2 and Keap1 expression in homogenates from MC exposed to NG (Ctr) or HG for different time periods (1–48 h). The upper part, representative western blot (**a**). The lower part, densitometric quantification of Nrf2 (**b**) and Keap1 (**c**) levels. The relative expression levels were normalized using actin. Values are the mean ± SEM (n = 7 per group) carried out in duplicate. NG, normal glucose; HG, high glucose. *p < 0.05 with respect to NG, ***p < 0.001 with respect to NG. Full-size blots are presented in Supplementary Fig. 3.

The Nrf2 expression levels from Müller cells extracts were analyzed by Western blot. Nrf2 immunoblotting revealed a single band of ≈ 68 kDa corresponding to the reported molecular weight. Nrf2 expression levels did not change in cells incubated with NG for different period times (Supplementary Fig. 3), so we averaged and normalized that as 100%. After 3 h incubation with HG, Nrf2 protein levels decreased by 48% ± 4.2; then, Nrf2 levels showed a progressive increase reaching control levels at 48 h (81% ± 2.5; Fig. 3a,b). Similarly, protein levels of Keap1 were significantly decreased in MC exposed to HG for 3 h (28% ± 6; Fig. 3a,c). Afterward, Keap1 protein levels gradually increased, reaching control levels at 48 h of incubation with HG (92% ± 11.1).

### Nrf2 subcellular localization

**Figure 4:**
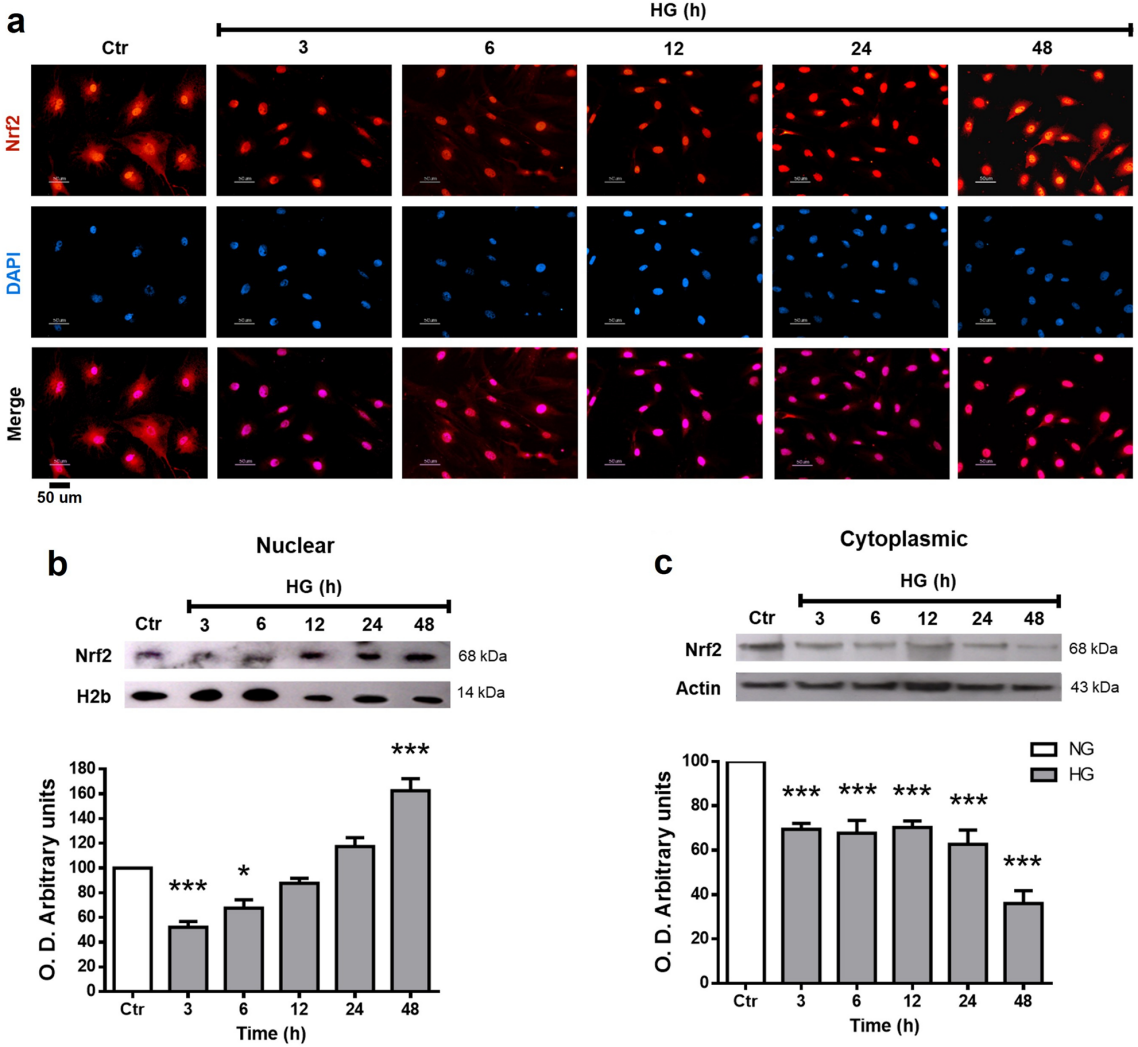
Nrf2 subcellular localization in MC. Cells were exposed to HG for the indicated time intervals. (**a**) Immunofluorescent localization of Nrf2 in MC. Blue marks nuclei (DAPI); Red, Nrf2-staining, and Pink, merge of blue and red indicating nuclear localization of Nrf2. (**b**) Nuclear and (**c**) Cytoplasmic Nrf2 expression in MC exposed to HG. The upper part, representative western blot of Nrf2. The lower part, quantification of the relative levels of Nrf2. The relative expression levels were normalized using actin (cytoplasmic) or H2b (histone 2b; nuclear). Values represent the mean ± SEM (n = 5 per group) carried out in duplicate. NG, normal glucose; HG, high glucose. Scale bar represents 50 µm. *p < 0.05 with respect to NG; ***p < 0.001 with respect to NG. Full-size blots are presented in Supplementary Fig. 4.

In response to oxidative stress, Nrf2 is translocated to the nucleus. We evaluated Nrf2 activation by immunofluorescence and Western blot of cellular fractions. Immunofluorescence studies showed Nrf2 distributed in the cell soma with a higher nuclear intensity in MC incubated with NG. Nrf2 labeling in the MC exposed to HG was located predominantly in the nuclear region (Fig. 4a). Similarly, Western blot analysis of the nuclear fraction obtained by differential centrifugation revealed relatively high Nrf2 levels in MC incubated in NG. Nrf2 nuclear levels were reduced at 3 h (48% ± 4.5) in MC exposed to HG, but after that, Nrf2 levels showed a progressive increase reaching levels higher than the NG at 48 h (161% ± 4; Fig. 4b). In contrast, cytoplasmic Nrf2 levels continuously decreased from 3 to 48 h (69% ± 2.7–34% ± 5.7) (Fig. 4c).

### High glucose decreases expression of antioxidant enzymes regulated by Nrf2

**Figure 5:**
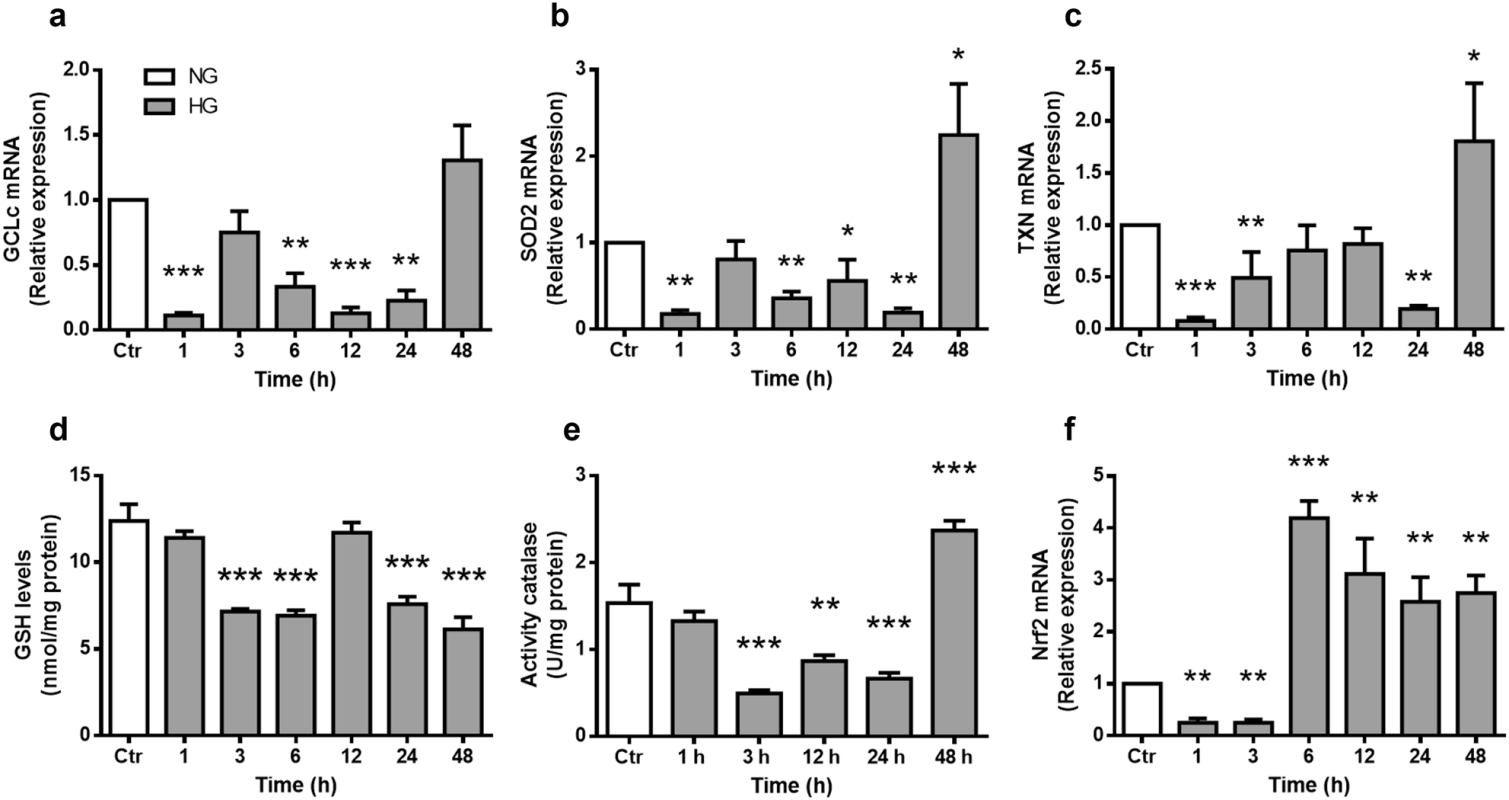
High glucose concentrations alter the expression of antioxidant enzymes and GSH levels. MC were exposed to HG for the indicated time intervals. mRNA levels of GCLc (**a**), SOD2 (**b**), TXN (**c**) and Nrf2 (f). The relative mRNA levels were normalized using ACT (actin). Values are the mean ± SEM (n = 4 per group) carried out in duplicate. (**d**) Glutathione (GSH) levels are expressed as nmol/mg protein. (e) Catalase activity is expressed as U/mg protein, as described in Methods. Values are the mean ± SEM (n = 5 per group) carried out in duplicate. NG, normal glucose; HG, high glucose. *p < 0.05 with respect to NG, **p < 0.01 with respect to NG, ***p < 0.001 with respect to NG.

Nrf2 binds to ARE DNA sequences, leading to transcription of antioxidant enzymes. We analyzed gene expression patterns of the Nrf2-regulated antioxidant enzymes Glutamate-Cysteine Ligase (catalytic subunit, GCLc) (Fig. 5a), Superoxide Dismutase 2 (SOD2) (Fig. 5b), and Thioredoxin (TXN) (Fig. 5c) in cultured MC. mRNA levels of these enzymes were considerably reduced after 1 h incubation within HG remaining low for up to 24 h incubation. Remarkably, mRNA expression of these enzymes was increased considerably at 48 h, reaching values higher than those in NG (Fig. 5a–c).

We also evaluated the effect of HG exposure on two of the main cellular antioxidants: catalase and glutathione (GSH). Catalase activity decreased in MC exposed to HG from 3 to 24 h (0.4 U/mg ± 0.03–0.6 U/mg ± 0.06), but significantly increased at 48 h (2.3 U/mg ± 0.1; Fig. 5d). GSH levels were reduced after a 3 h incubation with HG (7 nmol/mg ± 0.1) and remained low up to 48 h (5 nmol/mg ± 0.6; Fig. 5e).

Moreover, HG incubation for 1–3 h induced a considerable decrease in Nrf2 mRNA levels (0.3 ± 0.08–0.1 ± 0.06), but they were remarkably increased at 6 h (4.25 ± 0.3), reaching fourfold higher values than those in NG, and remained constant up to 48 h (2.8 ± 0.3; Fig. 5f).

### GSK3-β phosphorylation

**Figure 6:**
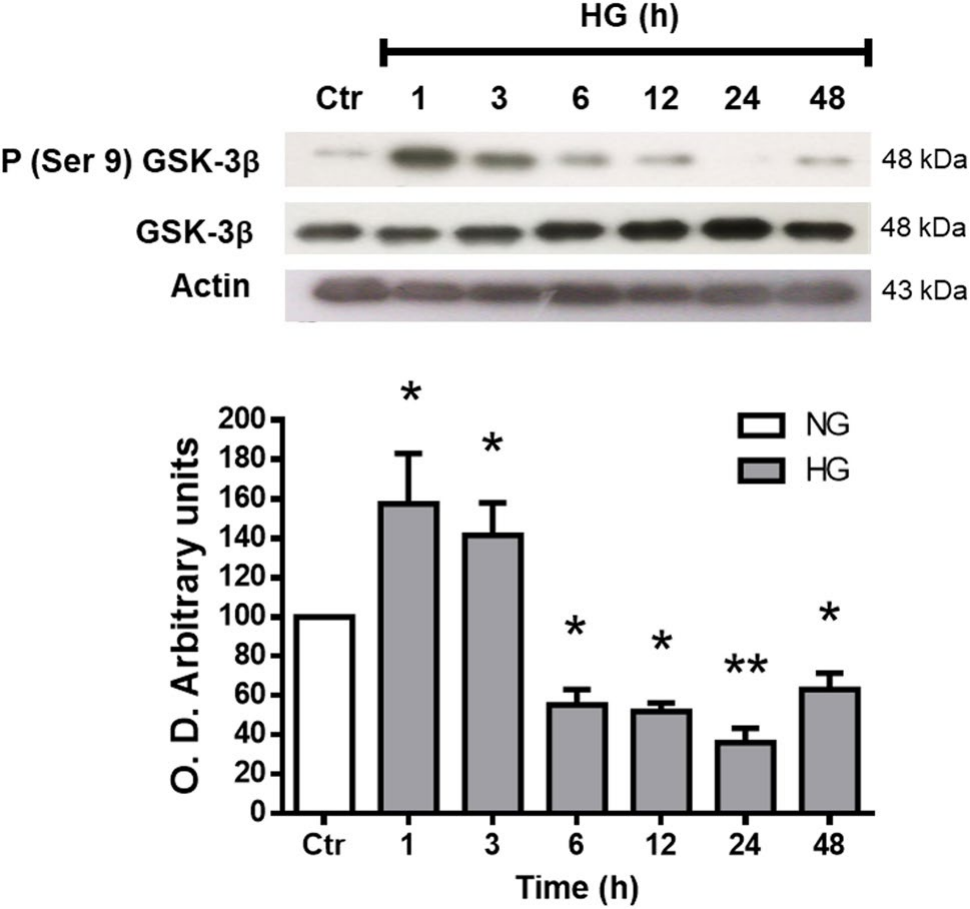
GSK3-β phosphorylation. Western blot analysis of phospho-GSK3-β (Ser 9) and GSK3-β expression in homogenates from MC exposed to NG (Ctr) or HG for different time periods (1–48 h). The upper part, representative western blot. The lower part, quantification of the expression levels. The relative expression levels were normalized with total GSK3-β. Values are the mean ± SEM (n = 4 per group) carried out in duplicate. NG, normal glucose; HG, high glucose. *p < 0.05 with respect to NG, ***p < 0.001 with respect to NG. Full-size blots are presented in Supplementary Fig. 5.

Because Gsk3 negatively regulates Nrf2 levels independently of Keap1, we evaluated Gsk3-β activation through its phosphorylation levels in Ser9. While total Gsk3-β levels did not change during all times and conditions studied, Gsk3-β phosphorylation levels were enhanced after 1 (151% ± 25.5) and 3 h (149% ± 16.4) HG incubation. After a 6 h incubation, Gsk3-β phosphorylation levels decreased (56% ± 7.7) from those of controls and remained low up to 48 h incubation (69% ± 8.3; Fig. 6).

### High glucose promotes the expression of inflammatory proteins in Müller cells

**Figure 7:**
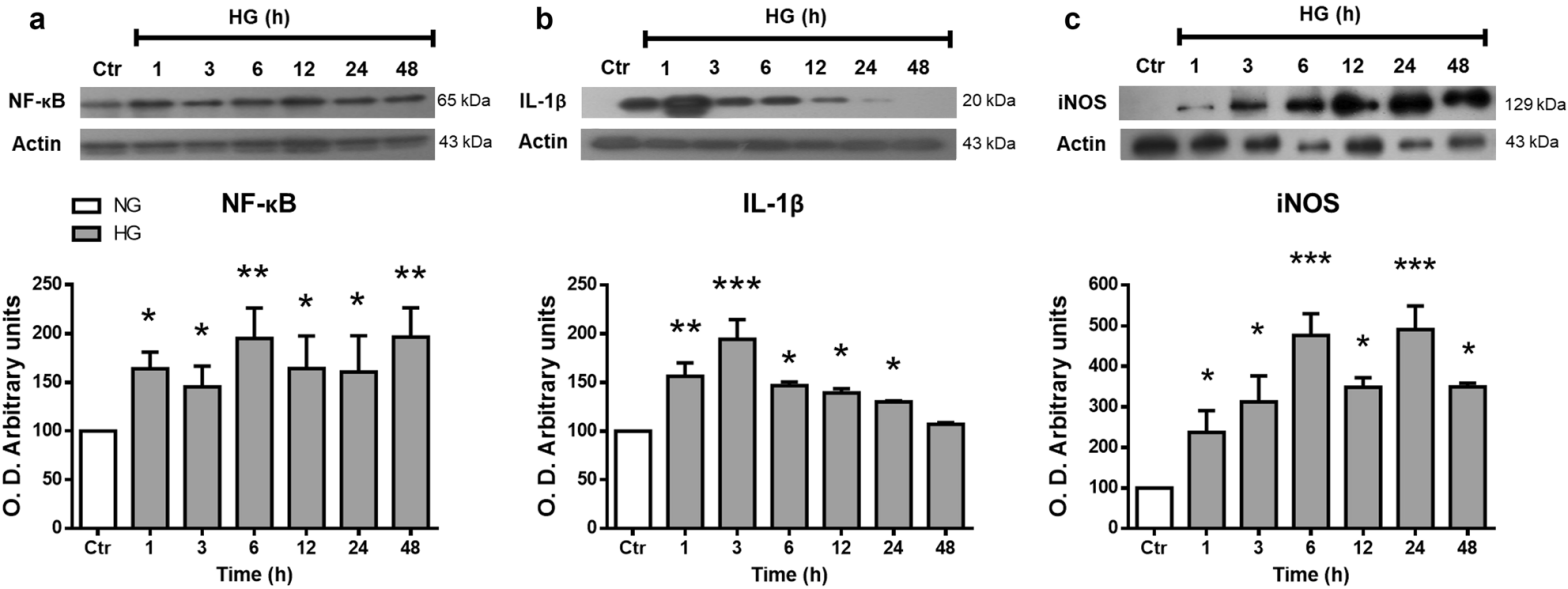
High glucose concentrations induced the expression of inflammatory proteins in MC. Western blot analysis of NF-κB (**a**), IL-1β (**b**), and iNOS (**c**) expression in homogenates from MC exposed to NG (Ctr) or HG for different time periods (1–48 h). The upper part, representative western blot. The lower part, quantification of the expression levels. The relative expression levels were normalized using actin. Values are the mean ± SEM (n = 5 per group) carried out in duplicate. NG, normal glucose; HG, high glucose. *p < 0.05 with respect to NG, ***p < 0.001 with respect to NG. Full-size blots are presented in Supplementary Fig. 6.

In addition to Keap1 and Gsk3-β, Nrf2 activity is negatively modulated by the transcription factor NF-kB. Therefore, we examined the effect of HG on its p65 subunit levels and of those of two of its target genes: interleukin-1β (IL-1β) and inducible nitric oxide synthase (iNOS) (Fig. 7). Canonical NF-kB subunit p65 levels were considerably increased since 1 h incubation in HG (171% ± 8.5; Fig. 7a). Also, HG caused a significant increase in the iNOS expression after 1–48 h incubation. Whereas IL-1β levels were elevated at 1 (154% ± 13.8) to 3 h (198% ± 20.3), and after that, its levels decreased progressively (Fig. 7b,c).

## Discussion

Under redox homeostasis, cells produce moderate ROS concentrations involved in cell signaling processes that are rapidly eliminated by antioxidant systems^34^. However, when the cellular redox state is altered by prolonged increases in ROS, surpassing the antioxidant defenses, cells suffer a state of oxidative stress leading to molecular damage^35,36^. Cells have an efficient antioxidant system, consisting of several antioxidant molecules that neutralize or eliminate oxidant molecules and indirect antioxidants that are redox-sensitive transcription factors. Among these factors, Nrf2 is considered the master regulator of the antioxidant response^37,38^. Oxidative stress has been strongly implicated in DR. Several studies have reported increases in ROS, decreases in antioxidant enzymes activities, and alterations in Nrf2 expression and function in the retina from long-term diabetic animals^39,40^.

The retina is a tissue composed of several cell types, and each cell type contribution to the development and progression of oxidative stress in DR has not been elucidated. Among them, glial Müller cells play a main role in maintaining neuronal function. Hyperglycemia induces the stress protein marker GFAP expression in MC. Besides, in vitro studies have shown occurrence of HG induced oxidative stress in different cell types, including MC^41–45^. There is also evidence that prolonged HG exposure increases hydrogen peroxide and nitric oxide levels in MC^46,47^. However, these studies evaluated single time points, preventing ascertainment of whether the redox imbalance is HG induced or is an after effect.

Several studies have reported that high glucose concentrations induce cell death in MC^30–32^. We observed a decrease in cell viability at 6 h of incubation with high glucose (Fig. 1a), however viability recovered values similar to the control in subsequent incubation times. The MMT assay is mainly associated with mitochondrial dehydrogenase activity, therefore its measurements can be affected by mitochondrial metabolism and by the activity of other intracellular dehydrogenases^48,49^, the decrease in cell viability at 6 h of exposure to high glucose could be due to transient changes in MC metabolism and not necessarily a reduction in cell viability. LDH (Fig. 1b) and TUNEL (Fig. 1c) assays did not show an increase in cell death. Studies that have reported changes in cell viability have used Müller glial cell lines (rMC-1 and MIO-M1)^31,32,50^ that might be more susceptible to the effect of high glucose than primary rat Müller glial cell cultures.

We analyzed the time course effect of HG incubation on ROS production in MC. As expected, ROS levels increased after a relatively short time incubation (6–12 h). We also found increase in RNS levels, and remarkably, they were elevated earlier than ROS (1 h) (Fig. 2). In agreement with the ROS increase, we observed that HG significantly reduced two main oxidant systems, GSH levels and catalase activity (Fig. 4), suggesting a redox homeostasis imbalance from early HG exposure. Nrf2 is known to regulate the basal and inducible transcription of various antioxidant enzymes, including catalase and enzymes regulating GSH synthesis^13^.

Therefore, we analyzed the expression of Nrf2 in MC. Western blot studies revealed the expression of Nrf2, which showed a substantial decrease at 3 h of HG incubation. After that, total Nrf2 protein levels progressively increased over time, being at 48 h, similar to those in NG. Under NG conditions, immunofluorescence studies demonstrated intense labeling of Nrf2 in the nucleus of MC, unlike other cell types in which Nrf2 localization in the absence of oxidative stress is restricted to the cytoplasm^51–53^. Thus, these results could indicate a high antioxidant capacity in MC. Proteomic studies have shown that MC have a high antioxidant capacity compared to different types of neurons in the retina^54^. Moreover, under HG conditions, immunofluorescence studies showed that Nrf2 is mainly located in the nuclear and perinuclear regions.

MC incubated in NG expressed relatively high Nrf2 levels in both nuclear and cytoplasmic fractions at all incubation times studied, in immunofluorescence experiments. Interestingly, incubation of MC within HG induced a transient decrease of Nrf2 in both nuclear and cytoplasmic fractions. While Nrf2 nuclear levels progressively recovered from 6 to 48 h, cytoplasmic levels decline. Surprisingly, the Nrf2 mRNA levels were also remarkably reduced after 1 and 3 h HG incubation; afterwards, mRNA levels were considerably higher, reaching values four-fold higher than those in NG (Fig. 5f).

The transient Nrf2 nuclear levels reduction may affect transcription of antioxidant genes. We found that mRNA levels of three Nrf2 target genes GCLc, SOD2, and TXN were reduced likewise at short HG exposure times. Unexpectedly, although Nrf2 mRNA and protein nuclear levels recovered after 6 h incubation in HG, its target genes expression and catalase activity took much longer to recover, only after 48 h of incubation with HG. Moreover, GSH levels remained lower, suggesting the occurrence of oxidative stress in spite of elevated Nrf2 nuclear levels. Despite this oxidative stress, we did not observe cell viability changes, suggesting a high capacity in MC to adapt to these conditions.

Multiple mechanisms control cellular Nrf2 function. The best-characterized Nrf2 regulation mechanism is mediated by its interaction with the Keap1-Cullin3-Rbx1 complex, which leads to Nrf2 ubiquitination and subsequent proteasomal degradation^55^. We found that Keap1 levels also decreased at short times of exposure to HG, but recovered in parallel to those of Nrf2, in agreement with Keap1 being a Nrf2 target gene^56^.

In addition to Keap1, Nrf2 contains a group of serine residues in its Neh6 domain that can be phosphorylated by GSK-3β. This phosphorylation creates a phosphodegron motif, which can be recognized by the β-TrCP-Cul1-Rbx1 E3 ubiquitin ligase complex, promoting the degradation of Nrf2^57,58^. We observed an increase of GSK-3β phosphorylation at short times upon HG exposure; therefore, consistent with the decrease of Nrf2 at early times. The decrease in GSK3-β phosphorylation over long periods could be due to a metabolic adaptation to HG exposure and appears not to affect Nrf2 levels. Moreover, the decrease in Nrf2 protein levels appears to be due to a decrease in its synthesis since we observed a transient decrease in its mRNA.

Various studies have identified that MC increase the secretion of pro-inflammatory protein levels in diabetic retinopathy^28,59,60^. NF-kB is a transcription factor that controls genes associated with inflammation, and its activation can negatively regulate Nrf2 expression and its target genes^61^. Interestingly, we found that incubation with high glucose increases the p65 canonical NF-kB subunit after short incubation times (1 h). Also, HG increases the levels of two NF-kB target genes: iNOS and IL-1b. The rapid iNOS increase may explained the RNS production observed at early incubation time with HG. These results also agree with early iNOS expression of iNOS in MC at early timepoint streptozotocin diabetes induction in rats^29^. These results are consistent with HG activating an inflammatory process, that in turn might negatively influences the expression of Nrf2 and causes the decrease of its target genes expression. Indeed, the p65 canonical NF-kB subunit competes with Nrf2 for the transcriptional coactivator CBP (CREB-binding protein) p300 complex^62,63^. Also, p65 promotes HDAC3 association with MafK, thus preventing heterodimer formation with Nrf2 and, as a consequence decreasing the expression of ARE-related genes^64,65^. It is noteworthy that Nrf2 controls its transcription, a fact that can also explain our results.

Therefore, our results are consistent with HG initially inducing an inflammatory process which could lead to oxidative stress. Interestingly, at longer incubation times, despite the Nrf2 recovery levels, there is a persistent redox state imbalance as shown by GSH levels, that may be part of an inflammatory response. Although viability was not affected, this imbalance might lead to alterations in cell homeostasis that can induce changes in the normal MC function, and this to homeostatic changes in other retinal cells, leading to retinopathy.

## Methods

### Müller Glia Culture

Retinal MC were isolated from postnatal (P 7–8 days) Long-Evans rats and were cultured using a protocol described by Hicks and Courtois^66^. Briefly, eyes were dissected out in Hank’s Ca^2+^ and Mg^2+^ free Balanced Salt Solution (H-4641 Sigma Aldrich) and incubated with trypsin (0.1%) at 37 °C for 30 min. Afterward, retinas were dissected in Dulbeco modified Eagles medium (D-2429 Sigma Aldrich) supplemented with 10% fetal bovine serum (FBS; Gibco), 2 mM glutamine, 100 U/ml penicillin, and 2.5 µg/ml amphotericin (DMEM-SFB). The retinas were mechanically dissociated by successive aspirations of the DMEM-SFB containing DNAse (100 U/ml). Suspended cells were counted using a Neubauer chamber and seeded in culture plates (6, 24, or 96 well) at a density of 160,000 cells/cm^2^. Cells were maintained in DMEM-FBS containing glucose (1 g/l) at 37 °C in a humidified atmosphere with 95% air/5% *CO*_2_. After 3 days in culture, the monolayer was vigorously rinsed to eliminate possible non-glial attached cells; medium was replaced every 3 days for fresh medium. Cells were used at approximately 90% confluency. The purity of Müller cell cultures was assessed by vimentin and glutamine synthetase immunofluorescence staining, specific markers of Müller glial cells. The percentage of the Müller glial cell purity is greater than 95% (Supplementary Fig. 1).

All procedures were in agreement with the Mexican Institutes of Health Research rules (DOF. NOM-062-Z00-1999) and with the Statement on the Use of Animals in Ophthalmic and Vision Research of the Association for Research in Vision and Ophthalmology. The protocol was approved by the Ethics Committee in Animal Experimentation of the Instituto de Fisiología Celular, UNAM (protocol number RSS43-14). All experiments were performed in accordance with relevant guidelines and regulations and ARRIVE guidelines.

### Incubation conditions

MC were incubated DMEM-SFB in the presence of normal (5 mM; NG) or high glucose concentrations (25 mM; HG) for different time periods (1–48 h). The culture medium was not changed during the treatment times. The cells treated with HG were compared with their respective control (NG) at the same times. The osmotic control medium was 5.5 mM glucose with 19.5 mM mannitol (Supplementary Fig. 2). The culture medium maintained high glucose concentrations during the different times studied (Supplementary Table 1).

### Western Blot

For Western Blot studies, MC were culture in 6-well plates, and after incubation with HG or NG, cells were rinsed with cold PBS and dislodged using a scraper. Cell samples were homogenized in RIPA lysis buffer (10 mM Tris-HCl pH 7.5, 158 mM NaCl, 1 mM EGTA, 10 mM Na_2_MoO_4_, 25 mM NaF, 1 mM phenylmethylsulfonyl fluoride, 1 mM Na_3_VO_4_, 1 mM EDTA, 2% Triton X-100, 0.2% SDS, and proteases-phosphatases inhibitors). The samples were incubated under constant shaking for 60 min at 4 °C and centrifuged at 17,000×*g* for 30 min at 4 °C. 25 µg of total protein for each sample were boiled in Laemmli’s sample buffer^67^ for 5 min and resolved by 10% SDS-PAGE together with molecular weight markers (Precision Plus Protein Kaleidoscope standards, Bio-Rad). The proteins were transferred to polyvinyl difluoride membranes (Millipore Corp.) according to standard techniques. The membranes were stained with Ponceau S to confirm that protein loading was the same in all lanes. Nonspecific protein binding sites were blocked with 5% nonfat milk in TBS-Tween (0.1% Tween 20, 20 mM Tris–HCl, and 136 mM NaCl, pH 7.6) for 3 h at room temperature, and then, membranes were incubated overnight at 4 °C with primary antibodies: Nrf2 (1:800; Abcam, ab137550), Keap1 (1:500; Santa Cruz, sc-33569), H2b (1:1000; Cell Signaling, 12364), Actin (1:3000, Abcam #ab-3280), iNos (1:1000, Abcam, ab95441), GSK3-β (1:1000; Cell Signaling, 9315), Phospho-GSK3 (1:800; Cell Signaling, 9323) or NF-*κ*B (1:1000, 8242, Cell Signaling), diluted with 0.25% BSA and 0.01% thimerosal in TBS-Tween buffer. The following day, membranes were washed three times with TBS-Tween and incubated for 3 h at room temperature with a secondary HRP-conjugated antibody (1:8000; Immobilon Western Chemiluminescent HRP Substrate, Millipore Corp.). The signal was detected using an enhanced Chemiluminescent HRP Substrate (Millipore Corp, Billerica, MA). The signals on Hyperfilm ECL (GE Healthcare Ltd.) were digitized with an Alpha DigiDoc RT (Alpha Innotech.) and analyzed using relative optical densities derived from a densitometry program (Alpha Ease FC Stand Alone, Alpha Innotech). The optical density of protein levels were normalized with their respective loading control using actin. The relative protein levels under the experimental conditions was obtained as the optical density of samples from cells incubated with HG compared to those of the control (NG) on the same Western blot.

### Protein content

Total protein content was determined according to Lowry et al.^68^ with a commercial assay kit (BioRad DC) using BSA as the standard.

### Subcellular fractionation

Cells were incubated in lysis buffer (50 mM HEPES (pH 7.5), 0.3 M sucrose, 1 mM EDTA, 1 mM PMSF, 1 mM Na3VO4, 0.1% Triton X-100, and proteases-phosphatases inhibitors) for 10 min on ice and homogenized. The lysates were centrifuged at 1000×*g* for 10 min at 4 °C. The supernatants were used as cytoplasmic fractions. The pellets (nuclei) were homogenized in nuclear buffer (50 mM HEPES (pH 7.5), 150 mM NaCl, 1 mM EDTA, 1 mM PMSF, 1 mM Na3VO4, 1% Triton X-100, 1% SDS, and proteases-phosphatases inhibitors) and centrifuged at 17,000×*g* for 15 min at 4 °C. The fractions were analyzed by Western blotting; immunoblot results for actin and histone H2b were used as markers for the cytoplasmic and nuclear fractions, respectively.

### Immunofluorescence

Cells were grown on coverslips and fixed with paraformaldehyde-(4% paraformaldehyde (W/V)—4% sucrose in 0.1 M PBS, pH 7.4) for 10 min at room temperature. Samples were rinsed three times for 5 min each with PBS. Cells were permeabilized with 0.4% saponin-PBS. Then, samples were blocked with 1% BSA-PBS for 1 h at room temperature. Samples were then incubated overnight at 4 °C with primary antibodies: Nrf2 (1:250, Abcam, ab137550); Keap1 (1:250, Santa Cruz, sc-33569); and Vimentin (1:250, Dako, Mo72529). Samples were washed three times and incubated with secondary antibody: Cy3-conjugated anti-rabbit (1:500, Chemicon) for 2 h at room temperature in a light-protected humidified box. Cell nuclei were stained with 300 nM DAPI (D4592, Sigma) for 10 min at room temperature. Samples were then washed with PBS. Coverslips were mounted onto glass slides with 79% glycerol (V/V in PBS) and stored in a light-protected container. For controls, primary antibodies were omitted. Samples were examined using a Nikon microscope (Nikon Corp., Tokyo, Japan) and photographed with a Nikon DXM1200 digital camera (Nikon Corp., Tokyo, Japan).

### Total RNA isolation and RT-PCR

Total RNA was extracted from the cells using the TRIZOL reagent (Invitrogen, Carlsbad, CA, USA) in accordance with the manufacturer’s protocol. cDNA was synthesized with M-MLV Reverse Transcriptase (Promega, M170A). Subsequently, Real Time-PCR (RT-PCR) was performed using SYBR Green I Master (Roche; 04 707 516 001) in the LightCycler 2.0 (Roche) according to the following thermal cycling conditions: the initial denaturation of one cycle at 95 °C for 10 s; followed by amplification with 50 cycles at 95 °C for 10 s, 62–69 °C for 10 s and 72 °C for 10 s; followed by the melting curve analysis with temperatures ranging from 60 to 95 °C. Primers used were (5′–3′): Thioredoxin (TXN), forward: ATGACTGCCAGGATGTTGCT, reverse: ACTCCCCAACCTTTTGACCC; Superoxide Dismutase 2 (SOD2), forward: GCTTGAATTGCTTGGACGCT, reverse: GCCCCAACACAGAGATGGAA; catalytic Glutamate-Cysteine Ligase (GCLc), forward: GAGCGAGATGCCGTCTTACA, reverse: TTGCTACACCCATCCACCAC; Actin B (ACTb), forward: ATGTGGATCAGCAAGCAGGA, reverse: AAGGGTGTAAAACGCAGCTCA and NRF2, forward: CACATCCAGACAGACACCAGT, reverse: CTACAAATGGGAATGTCTCTGC. Each biological sample was amplified in a technical replicate, and the average Ct (cycle threshold) value was used to determine the change in expression. Percentage change was calculated using the comparative Ct (2^−∆∆Ct^) method, where target mRNAs were normalized to the ACTb expression.

### Determination of ROS and RNS

The generation of intracellular reactive oxygen species was determined by two fluorescent markers sensitive to ROS: 2′,7′-dichlorodihydrofluorescein diacetate (H_2_DCFDA, which detects mainly hydrogen peroxide and superoxide), and dihydroethidium (DHE, specific to superoxide). The generation of reactive nitrogen species (RNS) was determined by 4-amino-5-methylamino2′,7′-difluorescein (DAF-FM, detects nitric oxide).

MC were cultured in 96-well plates. After treatment, the culture medium was eliminated, and the cells were rinsed with PBS and incubated with 25 µM H_2_DCFDA (Molecular Probes, Ref-C400) or DAF-FM (Life technologies, D-23842) in PBS for 30 min at 37 °C in a humidified atmosphere with 95% air/5% CO_2_. After incubation, cells were washed with PBS, and 100 µl PBS was added to each well. Fluorescence was measured with a multi-mode microplate reader (FlexStation; Molecular Devices) at 488 nm excitation and 535 nm emission. ROS levels were expressed as the percentage of relative fluorescence per µg protein (BCA method, Sigma, 500-0006). For microscopy studies, cells were cultured on coverslips and incubated under the same conditions.

To detect superoxide accumulation, the cells were incubated on coverslips with 10 µM DHE (Sigma, D7008) in DMEM medium for 30 min at 37 °C. The cells were then washed three times with PBS and mounted on glass slides and observed under a fluorescence microscope. The fluorescent quantification was determined using the calculation for the corrected total cell fluorescence (CTCF) = integrated density −  (area of select × mean fluorescence of background readings)^69^. For each image, three background areas were used to normalize against autofluorescence.

### GSH levels

Cells were washed with ice-cold PBS and scraped into ice-cold GSH buffer (1% sulfosalicylic acid, 0.1% Triton X-100 in 0.1 M phosphate buffer with 5 mM EDTA, pH 7.5). GSH levels in the cell extracts were measured by the DTNB-glutathione disulfide reductase recycling method described by Rahman et al.^70^. GSH concentration in the samples was determined using a standard GSH calibration curve, and the amount of GSH in the sample was expressed as nanomoles of GSH per milligram of protein.

### Catalase activity

Catalase activity was determined by measuring the decrease in H_2_O_2_. Cells were homogenized with 0.1% Triton in PBS and centrifuged at 17,000×*g* for 30 min at 4 °C. Supernatants were incubated with 5 mM H_2_O_2_ in PBS. The decrease of H_2_O_2_ was measured at 240 nm for 3 min at 30 s intervals, using a microplate reader (PowerWave HT, Biotek). Catalase activity in the samples was determined using a standard catalase calibration curve (Sigma, C1345; 0–150 u/ml). Catalase activity was expressed as units per milligram protein^71^.

### Cell viability

Cell viability was assessed by the 3-[4,5-dimethylthiazol-2-yl]-2,5-diphenyltetrazolium bromide (MTT) assay. After incubation with normal or high glucose, MTT (5 mg/ml-PBS) was added to each well and incubated for 4 h at 37 °C. The culture medium was then removed, and the insoluble precipitate was dissolved by adding 200 µl of dimethyl sulfoxide (DMSO). Optical densities at 510 nm were measured using a microplate reader (PowerWave HT, Biotek). Each experiment was performed in triplicate. Cell viability was calculated as the percentage of optical density respect to cells exposed to NG.

### Lactate dehydrogenase (LDH) assay

For LDH cell released determination, 500 µl of culture medium were collected at different incubation times and centrifuged. Supernatants were incubated with 120 µM NADH in 0.1 M of PBS for 3 min at room temperature, and the reaction was started with 2.3 mM pyruvate. The LDH activity of the samples was obtained by measuring the decreasing rate of NADH absorbance at 340 nm for 4 min using a microplate reader (PowerWave HT, Biotek). LDH activity was expressed as units per milliliter.

### TUNEL assay

For the TUNEL assay, we used the *in-situ* cell death detection fluorescein kit (Roche Diagnostic). Briefly, cells on coverslips were fixed as described for immunocytochemistry studies and permeabilized with 0.1% Triton X-100 in 0.1% citrate buffer for 2 min on ice, then incubated in TUNEL reaction mix at 37 °C for 1 h. Incubation without the deoxynucleotidyl transferase enzyme was conducted as a negative control. For positive control, cells were treated with DNAse I (0.1 mg/mL) for 15 min at 37 °C before the TUNEL reaction. Afterward, the coverslips were incubated with DAPI and mounted on glass slides with 79% glycerol. Samples were examined using a fluorescence microscope.

### Data presentation and statistics

All data are expressed as the mean ± SEM and were analyzed with GraphPad Prism 7 (GraphPad Software, La Jolla, C.) All experiments were performed a minimum of three times. Assessment of normal distribution of data was carried out with the Shapiro–Wilk test. Levene’s test was used to assess the homogeneity of variance between groups. Statistical significance was assessed by unpaired two-tailed t-test for comparison between two groups. One-way ANOVA with Tukey post hoc test, for multiple comparisons. A significant difference between the control and experimental group was defined with a *p* value of ≤ 0.05. The specific details of each experiment are provided in the corresponding figure legends.

## Supplementary Information

Supplementary Information.

## Data Availability

The datasets generated during and/or analyzed during the current study are available from the corresponding author on reasonable request.
