# Supplementary material for: High glucose concentrations induce oxidative stress by inhibiting Nrf2 expression in rat Müller retinal cells in vitro
Source: Sci Rep. 2022 Jan 24;12:1261. doi: 10.1038/s41598-022-05284-x (PMC8975969; doi:10.1038/s41598-022-05284-x)
Supplement: Supplementary file 1 — Supplementary Information. [file 41598_2022_5284_MOESM1_ESM.pdf]

High glucose concentrations induce oxidative stress by inhibiting Nrf2 expression in rat Müller retinal cells in vitro.

Jesús Silvestre Albert-Garay[1*], Juan Rafael Riesgo-Escovar[2] and Rocío Salceda[1,]

[1]Departamento de Neurodesarrollo y Fisiología, Instituto de Fisiología Celular, Universidad Nacional Autónoma de México CDMX, 04510, México.

[2]Instituto de Neurobiología, Campus UNAM Juriquilla, Universidad Nacional Autónoma de México, Querétaro, 76226, México.

[*]Address correspondence to Jesús Silvestre Albert-Garay: silvestre.alb@gmail.com

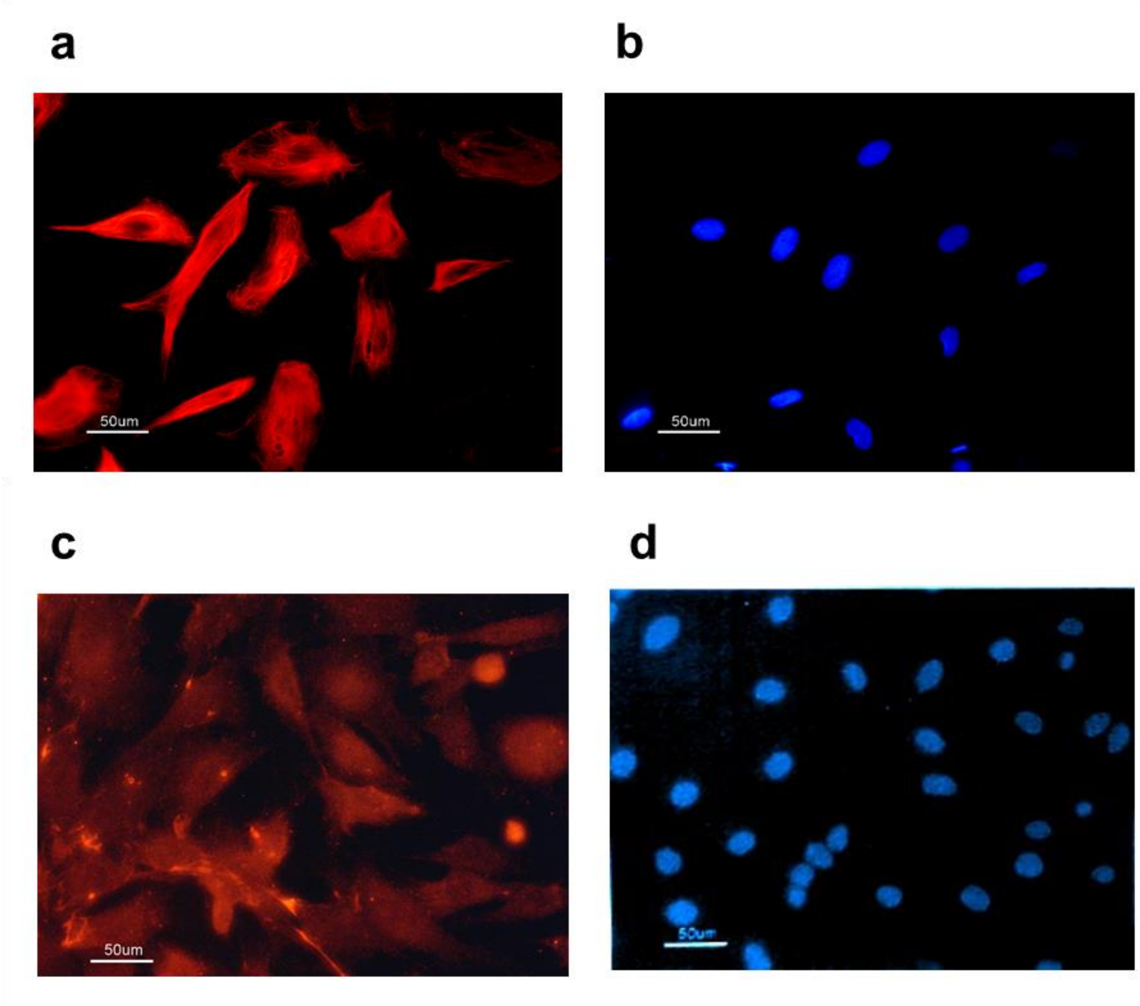

Supplementary Figure 1. Müller glial cells in culture express vimentin (a) and glutamine synthetase (c). Nuclei stained with DAPI (b and d). Scale bar represents 50 μm.

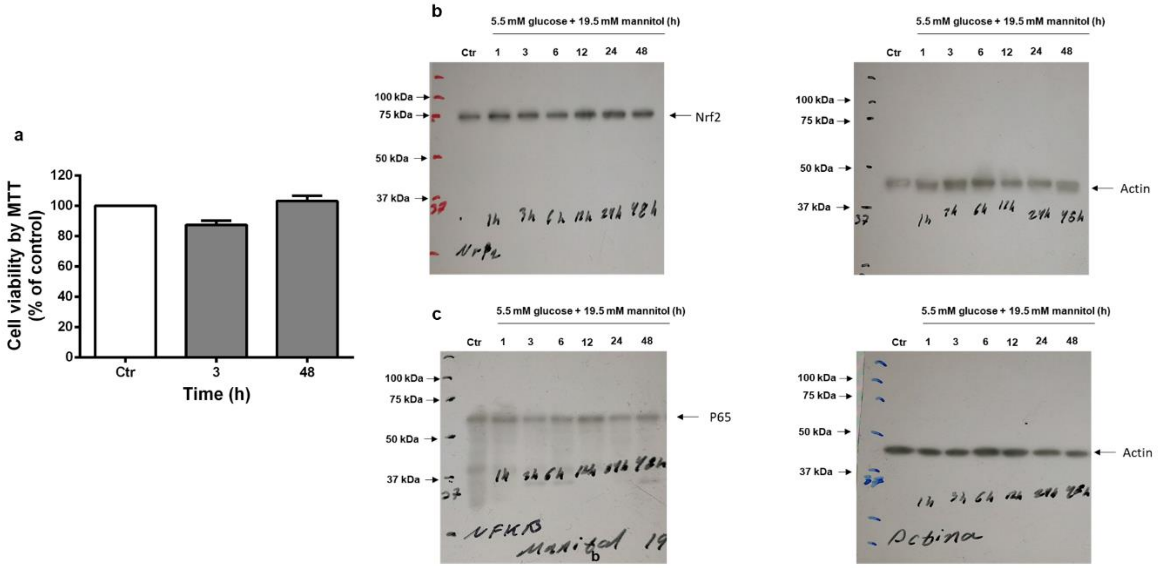

Supplementary Figure 2. Osmotic control with 5.5 mM glucose + 19.5 mM mannitol (a) Cell viability was determined by the MTT assay; viability was expressed as the percentage of optical density with respect to cells exposed to NG (100%). The osmotic condition did not affect the expression of Nrf2 (b) and P65 (c) during different periods of time (1-48 h). Data are expressed as the mean ± SEM of duplicate cultures and are representative of three independent experiments.

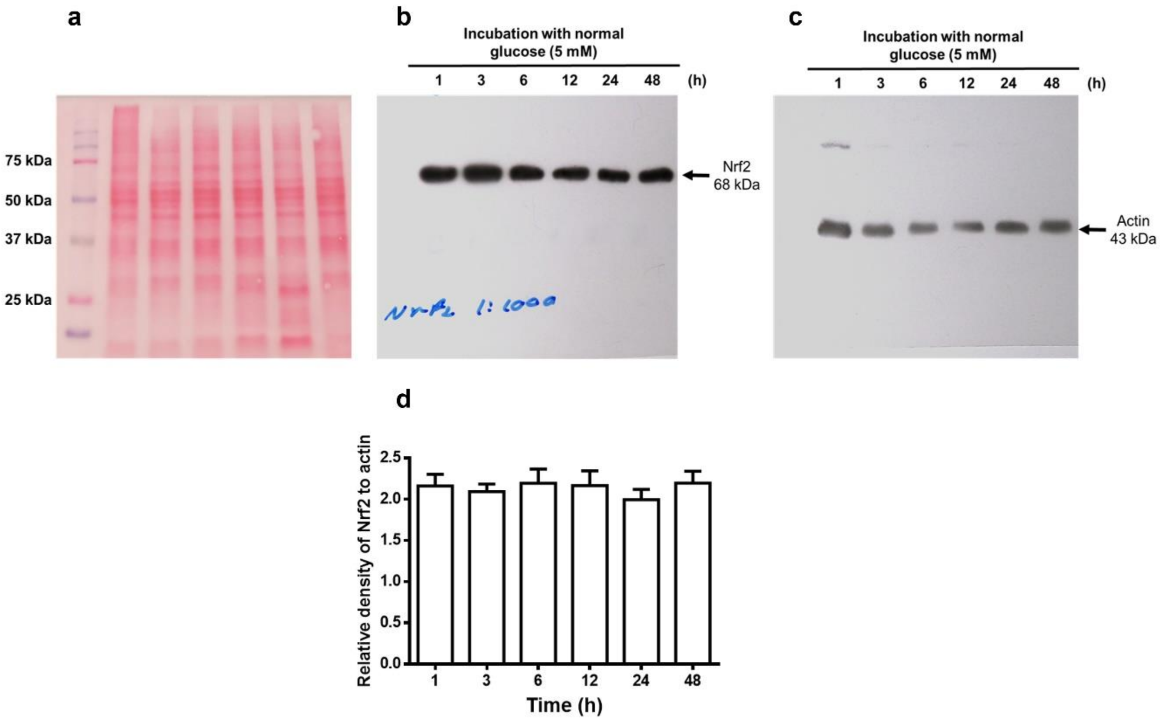

Supplementary Figure 3. Nrf2 expression in Müller cell cultures incubated with normal glucose. Ponceau S staining of the membrane (a) and blots using Nrf2 (b) and actin antibodies (c). The relative expression levels were normalized using actin (d). Müller cell cultures were incubated with normal glucose for different time periods (1- 48 h). Values are the mean ± SEM (n= 3 per group) carried out in duplicate.

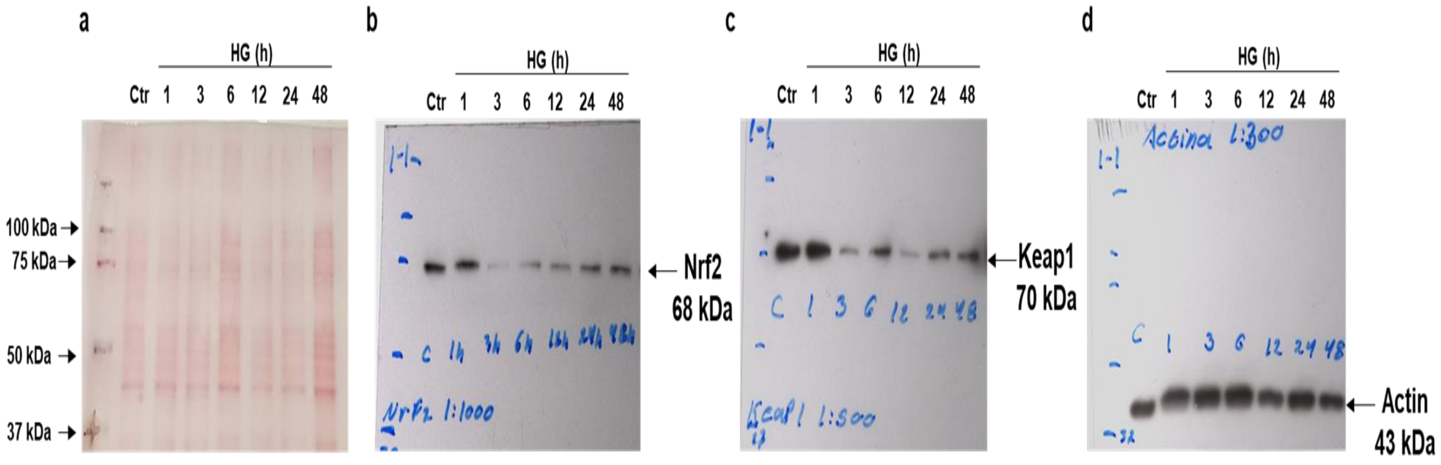

Supplementary Figure 4. Full size images of the cropped blots present in figure 3. Ponceau S staining of the membrane (a) and blots using Nrf2 (b), Keap1 (c) and actin (d) antibodies.

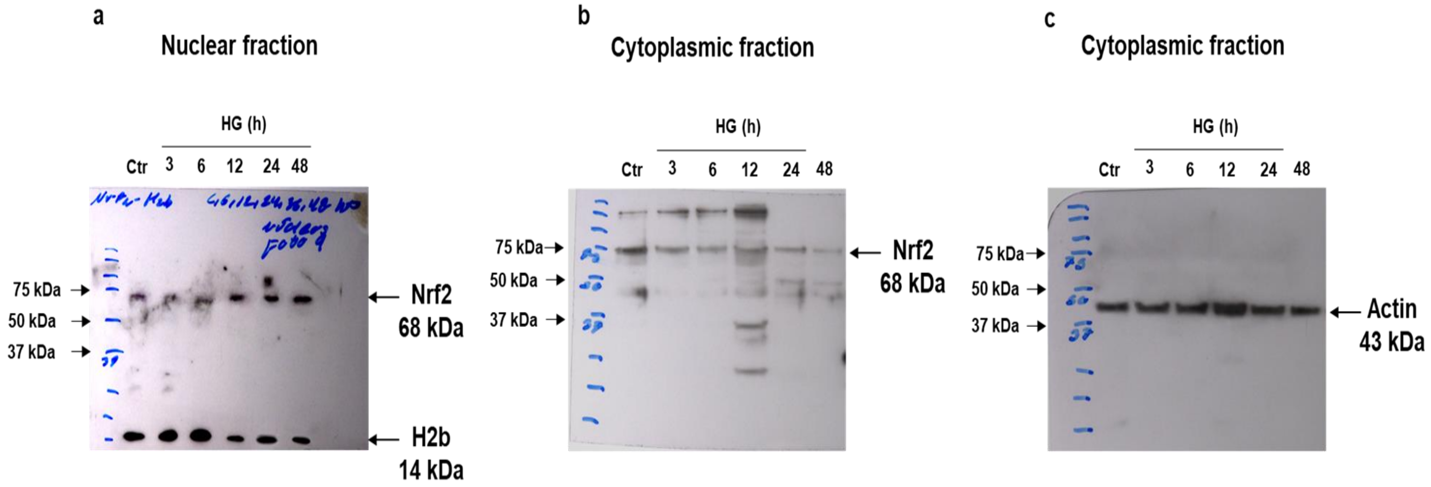

Supplementary Figure 5. Full size images of the cropped blots present in figure 4. Blot using Nrf2 and H2b antibodies in the nuclear fraction (a). Blots using Nrf2 (b) and actin (c) antibodies in the cytoplasmic fraction.

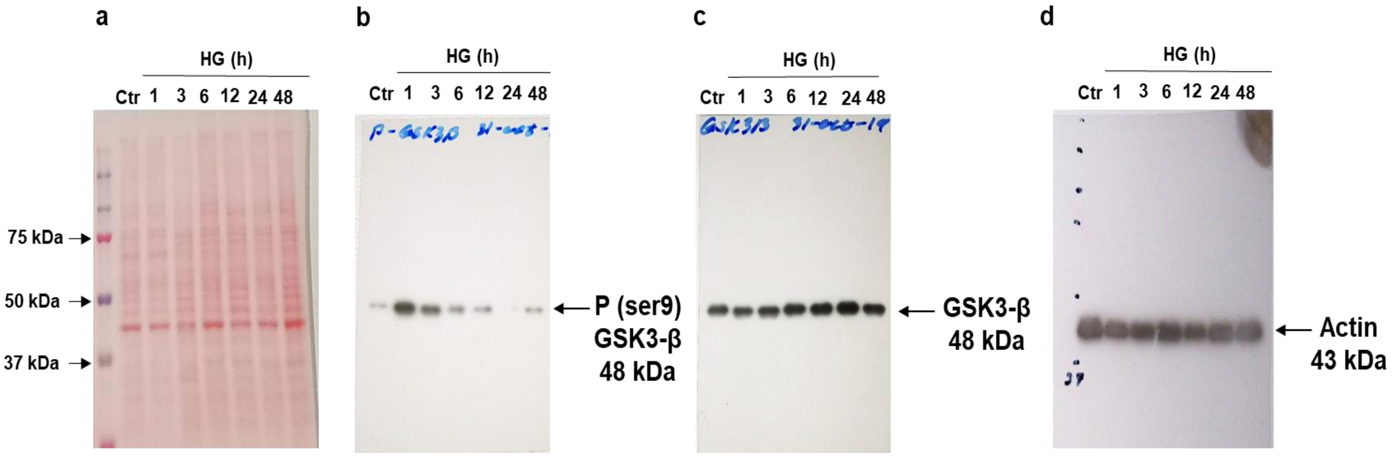

Supplementary Figure 6. Full size images of the cropped blots present in figure 6. Ponceau S staining of the membrane (a) and blots using P (Ser9) GSK-3β (b), GSK-3β (c) and actin (d) antibodies.

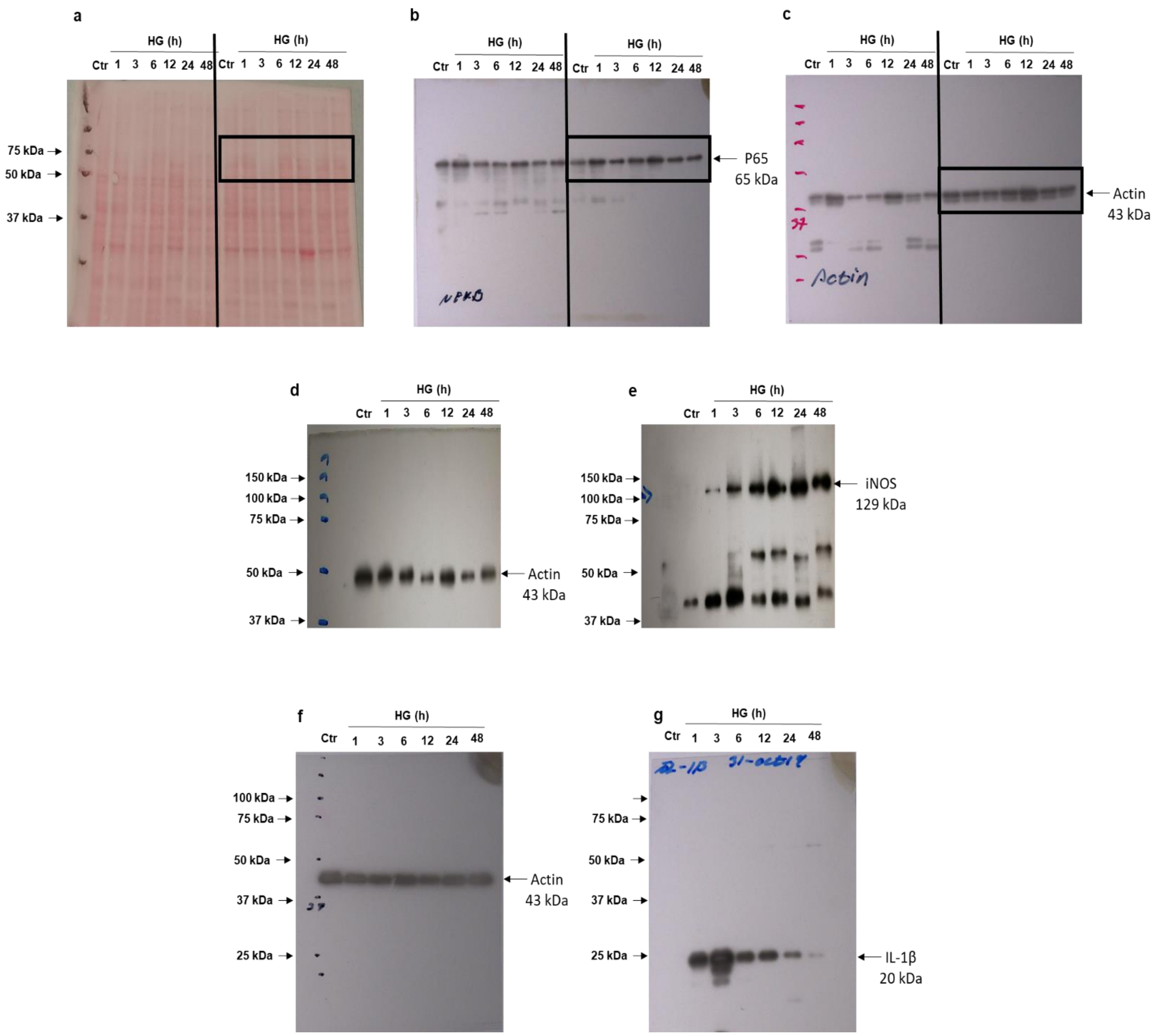

Supplementary Figure 7. Full size images of the cropped blots present in figure 7. Ponceau S staining of the membrane (a) and blots using P65 (b) and actin (c) antibodies. Blots using actin (d) and iNOS (e) antibodies. Blots using actin (f) and IL-1β (g). P65, INOS and IL-1β were immunodetected in different gels using actin as loading control.

Glucose levels in the culture medium							
Hours	0	1	3	6	12	24	48
Glucose mM	25.25 ±0.58	24.03 ± 0.42	23.3 ± 0.41	24.09 ± 0.19	22.03 ± 0.46	22.3 ± 0.48	22.1 ± 0.32

Supplementary Table 1. Glucose concentration in the culture medium during the different times studied. Values are the mean ± SEM (n= 5 per group)
